# Discovery and Characterization of a Novel Bipartite Botrexvirus From the Phytopathogenic Fungus *Botryosphaeria dothidea*

**DOI:** 10.3389/fmicb.2021.696125

**Published:** 2021-07-01

**Authors:** Mengmeng Yang, Wenxing Xu, Xiaoqi Zhou, Zuokun Yang, Yanxiang Wang, Feng Xiao, Yashuang Guo, Ni Hong, Guoping Wang

**Affiliations:** ^1^College of Plant Science and Technology, Huazhong Agricultural University, Wuhan, China; ^2^Zhengzhou Tobacco Research Institute of CNTC, Zhengzhou, China; ^3^State Key Laboratory of Agricultural Microbiology, Huazhong Agricultural University, Wuhan, China; ^4^Key Laboratory of Horticultural Crop (Fruit Trees) Biology and Germplasm Creation of the Ministry of Agriculture, Wuhan, China; ^5^Key Lab of Plant Pathology of Hubei Province, Wuhan, China

**Keywords:** *Botryosphaeria dothidea* botrexvirus 1, bipartite botrexvirus, *Alphaflexiviridae*, genome, *Botryosphaeria dothidea*, filamentous particles

## Abstract

In this study, we describe a novel positive, single-stranded (+ss) RNA mycovirus, named *Botryosphaeria dothidea* botrexvirus 1 (BdBV1), from a phytopathogenic fungus *Botryosphaeria dothidea* showing abnormal morphology and attenuated virulence. BdBV1 is phylogenetically related to Botrytis virus X (BotVX) and is the second potential member of the proposed genus *Botrexvirus* in the family *Alphaflexiviridae*. However, it differs from the monopartite BotVX in that BdBV1 possesses a bipartite genome comprised of two ssRNA segments (RNA1 and RNA2 with lengths of 5,035 and 1,063 nt, respectively). BdBV1 RNA1 and RNA2 encode putative RNA-dependent RNA polymerase (RdRp) and coat protein (CP) genes, which share significant identity with corresponding genes in both fungal and plant viruses. Moreover, open reading frames (ORFs) 2–4 of BdBV1 RNA1 shared no detectable identity with any known viral proteins. Immunosorbent electron microscopy (ISEM) analysis using an antibody against the virus CP generated *in vitro* revealed that BdBV1 is encapsidated in filamentous particles. A comparison of the biological effects of BdBV1 infection on symptoms and growth in isogenic lines of virus-free and virus-infected *B. dothidea* revealed that BdBV1 is probably involved in reduced growth and virulence of the host fungus. This study describes and characterizes a novel bipartite botrexvirus, which is closely related to uni- and multi-partite fungal and plant viruses and contributes useful information to a better understanding of virus evolution.

## Introduction

Mycoviruses (fungal viruses) are widely distributed in all major groups of fungi (Ghabrial et al., 2015). The vast majority of reported mycoviruses have double-stranded RNA (dsRNA) or linear positive single-stranded RNA (+ssRNA) genomes, and a few ssDNA or negative (−) ssRNA mycoviruses have also been described (Yu et al., 2010; Liu et al., 2014; Hao et al., 2018; Li et al., 2020). Although most mycoviruses are associated with latent infections, some can reduce the virulence of their hosts and can be regarded as potential alternative methods to control plant fungal diseases (Choi and Nuss, 1992). Moreover, the discovery of more novel mycoviruses has expanded our understanding of the origin, ecology, and evolutionary pathways of viruses (Ghabrial and Suzuki, 2009; Jia et al., 2017; Li et al., 2020; Sato et al., 2020).

Most dsRNA viruses are comprised of segmented genomes (Supplementary Table 1; Ghabrial et al., 2015), including the families *Partitiviridae* (two or three genomic segments, 1.4–2.3 kbp), *Chrysoviridae* (three to seven genomic segments, 2.4–3.6 kbp), and *Polymycoviridae* (four to eight genomic segments, 7.5–12.5 kbp) (Kanhayuwa et al., 2015; Jia et al., 2017; Sato et al., 2018). Conversely most + ssRNA mycoviruses possess non-segmented genomes but can, on occasion, contain one to three genome segments or, even in one case, Hadaka virus 1 (HadV1), which has an 11-segmented capsidless genome, which is phylogenetically closely related to the dsRNA polymycoviruses (Koonin et al., 2015; Sato et al., 2020). Some + ssRNA viruses express their proteins through the 3′-co-terminal sub-genomic RNAs (sgRNAs) such as the allexiviruses in the family *Alphaflexiviridae* and okaviruses in the family *Roniviridae*, while there are no reports concerning + ssRNA viruses displaying 3′-co-terminal genomic RNAs.

Here, we describe the characterization of a novel alphaflexivirus (tentatively named *Botryosphaeria dothidea* botrexvirus 1, BdBV1) from the phytopathogenic fungus *Botryosphaeria dothidea*, which contains a 3′-co-terminal bi-segmented genome distinct from non-segmented viruses that are harbored by known members in the same family, and are encapsidated in filamentous virions obviously larger than those of other members. BdBV1 potentially evolved from non-segmented alphaflexiviruses, and is closely phylogenetically related to both fungal and plant viruses. This study contributes useful information to better understand virus evolution in fungi.

## Materials and Methods

### Fungal Strains and Culture Conditions

*Botryosphaeria dothidea* strain L153 was isolated from canker-diseased pear stem tissue (*Pyrus communis* cv. Docteun Jule Guyot) in Dalian County, Liaoning Province, China. The virulent strain JNT1111 isolated from Jiangxi Province was used as control in this study (Zhai et al., 2014). Conidia were induced following inoculation of isolate L153 on detached pear leaves (*P. pyrifolia* cv. “Hohsui”) for longer than 7 days, prior to harvesting single conidia, which were separated and cultured further. Strain L153-29 is a single-conidium isolate progeny of L153. All strains were grown on potato dextrose agar (PDA) medium for 3–9 days at 25°C in darkness. Mycelial agar disks (5 mm) were preserved in sterilized 25% glycerol at −80°C.

### RNA Extraction and Reverse Transcription-PCR Detection

All fungal strains were grown on cellophane overlaid on the surfaces of PDA plates for 7 days, then mycelium was harvested and subjected to dsRNA extraction following a patented method (no. ZL201310072994.3) as described previously (Zhai et al., 2019). DsRNA preparations were separated by 1.0% (*w*/*v*) agarose gel electrophoresis and visualized by staining with ethidium bromide. Total fungal RNA samples were extracted using TRIzol reagent, according to the manufacturer’s instructions (ThermoFisher Scientific, Inc., Waltham, MA, United States). For reverse transcription-PCR (RT-PCR) detection of different mycoviruses in fungal strains, first-strand cDNA was synthesized using Moloney murine leukemia virus (M-MLV) transcriptase (Promega Biotech Co., Ltd.) using total RNA as template and amplified using Taq DNA polymerase (TaKaRa, Dalian, China).

### Deep Sequencing Analysis and Amplification of the Viral Genomic RNAs

The total RNA of strain L153 was used as a template for deep sequencing analysis. Sequencing was performed on the Illumina HiSeq XTen sequencing machine with a paired-end 150-bp setup (Biomarker Biology Technology Ltd., Company, Beijing, China). Ribosomal RNA (rRNA) was removed from fungal total RNA using an Epicentre Ribo-ZeroTM kit (Epicentre, Madison, WI, United States), then subjected to RNA-seq. The cDNA library was constructed using TruSeq RNA Sample Prep Kit v2 (Illumina, San Diego, CA, United States). The raw RNA reads were cleaned by removing adapter sequences, and low-quality reads with more than 5% Ns or with 20% base quality values (Q20) less than 20 were filtered using FASTP version 1.5.6. All clean reads were *de novo* assembled using Velvet version 14 1.2.08 with a *k*-mer value = 17 (Zerbino and Birney, 2008). Assembled reads were screened for sequence identities against the National Center for Biotechnology Information (NCBI) databases using the BLAST program.^1^

First, 14 sets of oligonucleotide primers were designed based on the contig sequence assemblies and used to confirm the viral sequences through RT-PCR amplifications. The 5′-terminal sequences of the viral RNA were determined by the use of a commercial kit (TaKaRa 5′-Full RACE Kit, Dalian, China) following the manufacturer’s instructions. The 3′-terminal regions were determined by using a universal primer M4-T (5′-GTTTTCCCAGTCACGACGATTTTTTTTTTTTTTTT-3′) for reverse transcription, then primer M4 (5′-GTTTT CCCAGTCACGAC-3′) and viral specific primers were used for PCR amplification. All PCR products were cloned and sequenced as previously described (Yang et al., 2019).

### Biological Properties of Fungal Strains

Growth rates and morphology of fungal strains were estimated by culturing freshly grown mycelial agar plugs (diameter, 5 mm) on PDA medium (diameter, 9 mm) in quadruplicate at 25°C in the dark for 10 days (Yang et al., 2019). The colony morphology and mycelial diameter were estimated every 24 h. The virulence of fungal colonies was assessed on detached pear fruits (*P. Brettschneider* cv. “Huang guan”). All pear fruits were selected and share a consistent appearance and texture. Fungal strains were inoculated on wounded mature pear fruits using colonized agar plugs (5 mm in diameter) and incubated at 23–25°C. All inoculations were repeated four times. The lesions were measured and photographed at 7 days post-inoculation. To observe the morphology of hyphal tips, all *B. dothidea* strains were cultured on PDA for 3–7 days, and hyphal tips of each strain were observed using a microscope and photographed using a digital camera (Eclipse 55i; Nikon).

## Virus Purification and Peptide Mass Fingerprinting Analysis of Viral Proteins

*Botryosphaeria dothidea* botrexvirus 1-infected (L153) and BdBV1-free (L153-29) strains were used for virus purification. Approximately 50 g of mycelia cultured on cellophane membranes overlaid on PDA for 7–9 days were harvested and ground into fine power in liquid nitrogen. The resulting powder was homogenized in a conical flask with 200 ml of phosphate buffer (PB, 0.1 M, pH 7.4 containing 0.1% β-mercaptoethanol) and centrifuged at 10,000 × *g* for 15 min two times to remove cellular debris. The final supernatant was further ultracentrifuged at 100,000 × *g* for 3 h (Optima LE-80K; Beckman Coulter, Inc., Brea, CA, United States). The precipitates were resuspended in 0.05 M PB buffer, and the supernatants were centrifuged in sucrose density gradients (10–60%) at 70,000 × *g* for 3 h. The purified virus particle was negatively stained with 2% (w/v) uranyl acetate on carbon-coated 230-mesh copper grids and observed with a transmission electron microscope (TEM; H7650; Hitachi and H-7000FA; Hitachi).

Proteins extracted from each sucrose fraction were detected by 12% (w/v) SDS-PAGE with 25 mM *Tris*-glycine and 0.1% SDS. The gels were visualized by staining with Coomassie brilliant blue R-250 (Bio-Safe CBB; Bio-Rad, Irvine, CA, United States), then protein bands on the gels were individually excised and subjected to peptide mass fingerprinting (PMF) analysis at Sangon Biotech (Shanghai, China) Co., Ltd.

### Ethics Statement

The animal study was reviewed and approved by the Research Ethics Committee, Huazhong Agricultural University, Hubei, China, and carried out in accordance with the recommendations in the Guide for the Care and Use of Laboratory Animals from this Committee.

### Expression of Fusion Protein and Polyclonal Antibody Production

Total RNA extracted from strain L153 was used as template for the RT-PCR amplification of ORF5 using a specific primer pair ORF5-*Bam*HI-F (5′-CGCGGATCCATGTTCCCTACGCTTTCGAAAATGGATCC-3′) and ORF5-*Xho*I-R (5′-CCGCTCGAGCCATCCGATTTGTG GGCGGTTGGGGACCCGTT-3′). PCR amplicons were purified and ligated into expression vector pGEX-KG. The recombinant plasmids (pGEX-KG-ORF5) were transformed into *Escherichia coli* Rosetta (DE3) cells. Fusion proteins in *E. coli* were induced with 1.0 mM isopropyl-thio-β-D-galactoside (IPTG) in a shaker at 200 rpm at 28°C for 8–10 h. Proteins were separated by SDS-PAGE and visualized by soaking in 0.5 M KCl following a previously reported method (Vaira et al., 1996). Recombinant protein bands on the gel were excised and used to raise polyclonal antibody (PAb-P5) generation following four injections into Japanese white rabbits (weight > 2.0 kg, provided by the Laboratory Animal Centre, Huazhong Agriculture University, Hubei, China) following previously described methods (Tunitskaya et al., 2016).

### Immunoblotting, Indirect Enzyme-Linked Immunosorbent Assays, and Immunosorbent Electron Microscopy Analysis

Following sucrose gradienting of purified virus, isolated from virus-infected, fungus strain L153 and a separate equivalent extract from virus-free, fungal strain L153-29, individual fractions were separated by 12% SDS-PAGE prior to immunoblotting as previously described by Song et al. (2011). Indirect enzyme linked immunosorbent assays (ELISA) were performed as previously described with PAb-P5 at antibody dilution ranging from 500 to 512,000 (Wu et al., 2014). Purified virus preparations were also analyzed by immunosorbent electron microscopy (ISEM) analysis with PAb-P5 at a dilution of 1:4,000 or 1:2,000 (Agranovsky et al., 1995).

### Sequence and Phylogenetic Analyses

Sequences obtained from clones were assembled using SeqMan software (version 7.1.0, DNASTAR Inc., Madison, WI, United States) (Burland, 2000). Viral sequence data were analyzed using DNAMAN software package (DNAMAN, version 6.0; Lynnon Biosoft, Montreal, QC, Canada). Conserved protein domains were predicted by the NCBI conserved domain database (CDD) (see text footnote 1) (Marchler-Bauer et al., 2017). Multiple sequence alignments of amino acid (aa) sequence were performed using MAFFT.^2^ The resulting data were presented using GeneDoc software (version 2.7.0). Phylogenetic trees were constructed using the maximum likelihood (ML) method in Molecular Evolutionary Genetics Analysis (MEGA) software 7 with bootstrap values estimated by 1,000 replicates (Kumar et al., 2016). Reference sequences of viruses used for comparative analyses were obtained from the NCBI database.^3^

## Results

### Mycoviruses Identified in *Botryosphaeria dothidea* Strain L153

To investigate the viruses in *B. dothidea* strain L153, total RNA was extracted and subjected to high-throughput sequencing, and the resulting reads were assembled, and seven contigs ranging in size from 1,088 to 5,026 nt were obtained. BLASTx searches of these contig sequences in NCBI database revealed four novel + ssRNA viruses and one known dsRNA virus (*Botryosphaeria dothidea* partitivirus 1, BdPV1). Of these + ssRNA mycoviruses, three contain open reading frames (ORFs) sharing the highest identities with RNA dependent RNA polymerase (RdRp) sequences of the *Plasmopara viticola*-associated narnavirus 16 (PvaNV16, 58.6%, *E*-value = 0, and 37.9%, *E*-value = 5e-108) and *Plasmopara viticola*-associated narnavirus 9 (PvaNV9, 55.3%, *E*-value = 0) belonging to the family *Narnaviridae*, and tentatively named *Botryosphaeria dothidea* narnavirus 2–4 (BdNV2–4); the remaining one shares the highest identity (51.5%, *E*-value = 0) with that of Botrytis virus X (BotVX), the only member in genus *Botrexvirus* of the family *Alphaflexiviridae*, and tentatively named BdBV1. BdPV1 RdRp shares the highest identity of 99.5% (*E*-value = 0) with that of the isolate LW-1 from *B. dothidea*, whose dsRNAs were confirmed by agarose gel analysis of the dsRNA extraction as expected sizes (Supplementary Figure 1A). The partial genome nucleotide sequences of BdNV2–4 were further confirmed using the specific primers designed based on the contigs obtained (Supplementary Figure 1B) and deposited in the GenBank database under the accession numbers MT103580 to MT103582, respectively.

### Genomic Organization of BdBV1

**FIGURE 1:**
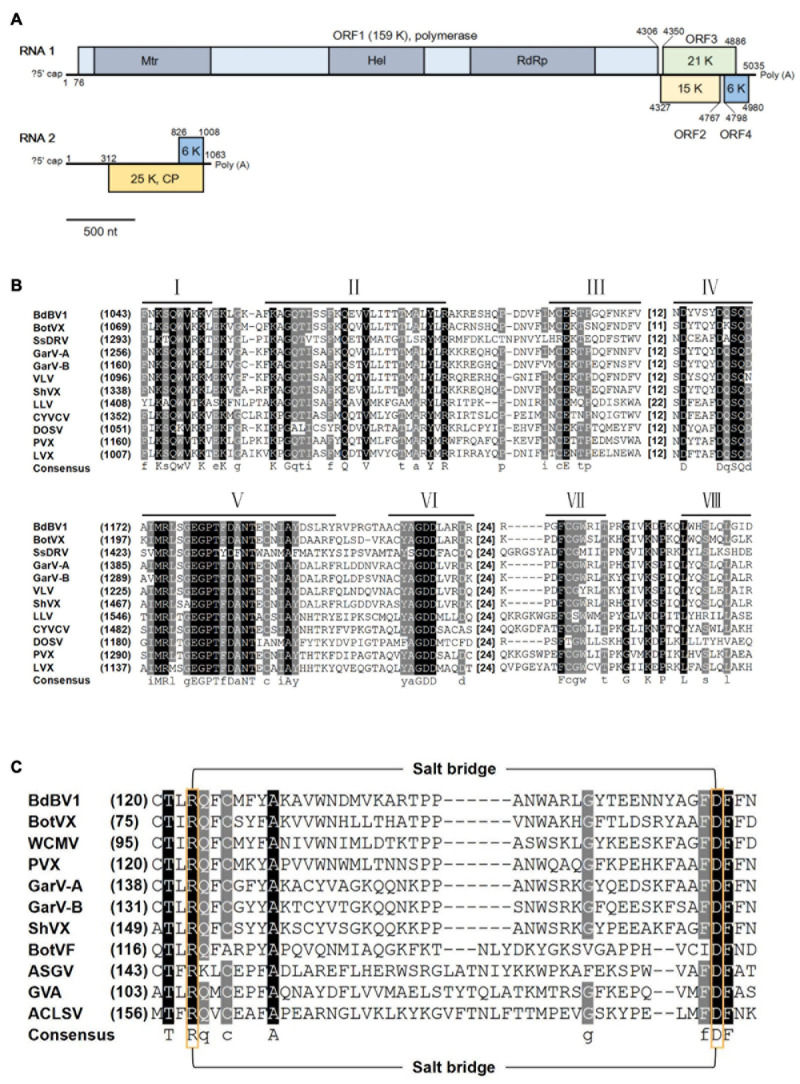
Genomic properties of *Botryosphaeria dothidea* botrexvirus 1 (BdBV1). **(A)** Schematic representation of the genomic organization of BdBV1. Different proteins are represented by different colored boxes, three conserved domains in ORF1 are indicated by darker areas. **(B)** Multiple alignment of the region corresponding to RdRp domain of BdBV1 and other selected alphaflexiviruses. Eight core RdRp motifs are indicated with lines above. The starting amino acid (aa) position and spacing between motifs are indicated. **(C)** Multiple alignment of the CP aa sequences of BdBV1 and other flexuous rod-shaped viruses potentially involved in salt-bridge formation. The conserved positively (Arg) and negatively (Asp) charged residues are indicated by yellow boxes.

The full-length sequence of BdBV1 was determined by overlapping RT-PCR amplification using specific primers designed based on contig sequences together with RACE. BdBV1 contains two 3′-co-terminal + ssRNA segments (RNAs1 and 2) 5,035 nt (RNA1) and 1,061 nt (RNA2) in length excluding poly(A) tails and both have been deposited in the GenBank database under accession numbers MT103578 and MT103579, respectively. The 5′-terminal sequences of RNA1 and RNA2 contained no conserved sequences. RNA1 has a GC content of 62.5%, contains four putative ORFs (designated as ORF1–4), and 5′- and 3′-untranslated regions (UTRs) 210 and 417 nt, respectively, in length. ORF1 is 4,230 nt in length (nt position: 77–4,306), putatively encoding a RdRp (P1) of 1,408 aa with a deduced molecular weight (MW) of 159 kDa (Figure 1A). BLASTp analysis showed that P1 shared the highest identity of 57.4% (*E*-value = 0, coverage 48%) with the RdRp encoded by garlic virus A (GenBank accession no. AGG13288.1) and significant identity of 51.5% (*E*-value = 0, coverage 96%) with the replicase encoded by BotVX (GenBank accession no. NP_932306.1, Howitt et al., 2006; Kreuze et al., 2012), and lower identities with RdRps encoded by other plant viruses belonging to the same family. CDD database search (Marchler-Bauer et al., 2017) of P1 showed that it contained a viral methyltransferase (Mtr, accession no. pfam01660, *E*-value = 4.65e-65) domain, an RNA helicase (Hel, accession no. pfam01443, *E*-value = 1.55e-47) domain, and an RdRp domain (accession no. cl03049, *E*-value = 1.32e-05) (Figure 1A). Alignment of the aa sequences of the P1 RdRp domain with those of other related viruses revealed the presence of eight motifs, including a GDD motif, which is a typical feature of viral RdRps (Figure 1B; Ghabrial, 1998). BdBV1 ORF2 (nt position: 4,327–4,767) located downstream of ORF1 following a short intergenic non-coding region (IG-NCR), 20 nt in length, putatively encoding a protein (P2) of 145 aa with a calculated MW of 15 kDa (Figure 1A). BLASTp searches of P2 revealed no significant identity with other known proteins, but a PRK11907 super family domain (accession no. cl36084, *E*-value = 1.01e-05) was following a CDD search (Liu et al., 1986). BdBV1 ORF3 (178 aa, nt position: 4,350–4,886) encodes a putative protein (P3) with an expected MW of 21 kDa and shares the highest identity of 29.1% (*E*-value = 8.4, coverage 63%) with a hypothetical protein (accession no. XP_024513943.1) of *Cryptococcus neoformans*, which contains an SMC prok_A super family domain [structural maintenance of chromosomes (SMC), accession no. cl37070, *E*-value = 1.22e-03] (Cobbe and Heck, 2004). BdBV1 ORF4 (nt position: 4,798–4,980) is 59 aa in length and encodes a putative protein with a MW of 5 kDa, which shares no detectable identity with other known proteins.

*Botryosphaeria dothidea* botrexvirus 1 RNA2 has a GC content of 63.7%, with a long 5′-UTR of 312 nt and a short 3′-UTR of 55 nt and contains one ORF (ORF5, nt position: 313–1,008) encoding a putative protein (P5) with an expected MW of 25 kDa. BLASTp analysis of P5 showed that it shares the highest identity of 48.8% (coverage 52%, *E*-value = 9e-31) with the coat protein (CP) of the plant virus white clover mosaic virus (WCMV, accession no. BAV16907.1), and has an identity of 42.5% with the CP of mycovirus BotVX (coverage 79%, *E*-value = 4e-34). A CDD database search of BdBV1 P5 revealed that it contains a Flexi_CP super family protein domain (accession no. cl02836, *E*-value = 5.71e-45) commonly found in the of potexviruses and carlaviruses (Majumder and Baranwal, 2011). In addition, alignment of BdBV1 ORF5 and CP sequences of other related viruses revealed the presence of a putative salt bridge (Figure 1C), which was reported as the hydrophobic core in flexuous, rod-shaped ssRNA plant viruses (Dolja et al., 1991).

### Phylogenetic Analysis and Genomic Structure Alignment

**FIGURE 2:**
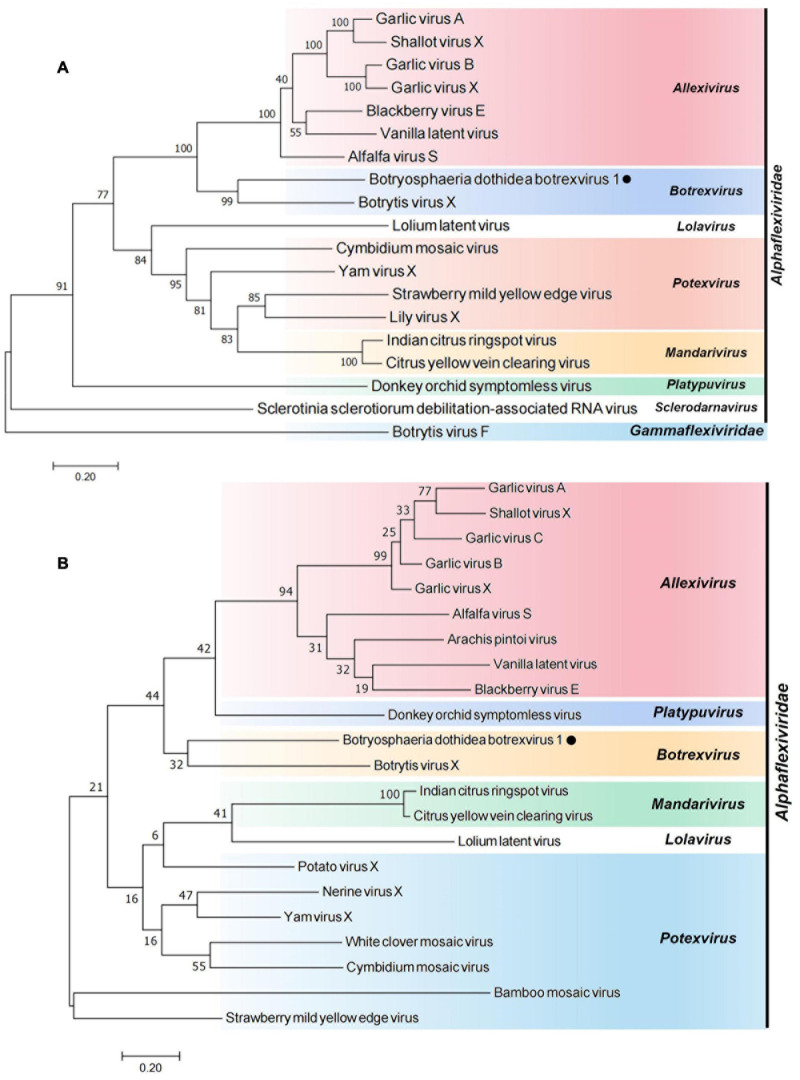
Phylogenetic relationships of BdBV1 with other related viruses. **(A)** Tree created based on the replicase sequences. **(B)** Tree created based on the CP sequences. BdBV1 is indicated by a black circle, genera within the *Alphaflexiviridae* are indicated in different colors.

To study the phylogenetic relationship of BdBV1 with other members in the family *Alphaflexiviridae*, phylogenetic trees were created based on the complete replicase and CP aa sequences. Both RdRp (Figure 2A) and CP (Figure 2B) phylogenetic trees indicated that BdBV1 clusters with mycovirus BotVX which belongs to genus *Botrexvirus*. Thus, BdBV1 is considered to be a novel member of genus *Botrexvirus* (Howitt et al., 2006; Rosario et al., 2012).

A phylogenetic tree was constructed based on the RdRp aa sequences of BdNV2–4 and other related virus members. Phylogenetic analysis revealed that three narnaviruses all formed a well-supported clade with other members of the genus *Narnavirus* and independent from other genera of the family *Narnaviridae* (Supplementary Figure 2). Thus, BdNV2, 3, and 4 may be three novel narnaviruses.

### Biological Tests of *Botryosphaeria dothidea* Botrexvirus 1-Infected and -Free Strains

**FIGURE 3:**
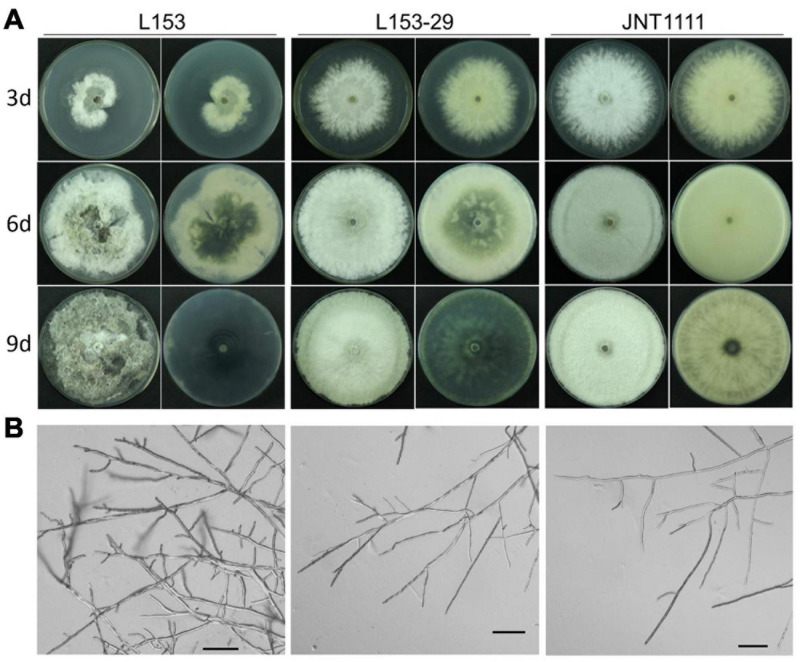
Colony and hyphal characteristics of *B. dothidea* strains. **(A)** Colonial morphology of strains L153, L153-29, and JNT1111 growing on potato dextrose agar (PDA) at 25°C for 3, 6, and 9 days. The front view and rear view were indicated by left and right sides, respectively. **(B)** Hyphal tips of *B. dothidea* strains. Scale bar, 100 μm.

L153 revealed that the virus-infected strain exhibited an abnormal colony phenotype with irregular margins and produced more pigment as compared with the standard *B. dothidea* strain JNT1111 (Figure 3A). To explore whether these abnormal properties are related to virus infection, colonies generated from individual conidia of strain L153 were investigated for virus presence by dsRNA extraction and RT-PCR amplification of virus-specific amplicons. The results showed that a sub-strain (designated as strain L153-29) contains no BdBV1 but did contain the other four viruses (e.g., BdNV2–4 and BdPV1) (Supplementary Figure 1).

**FIGURE 4:**
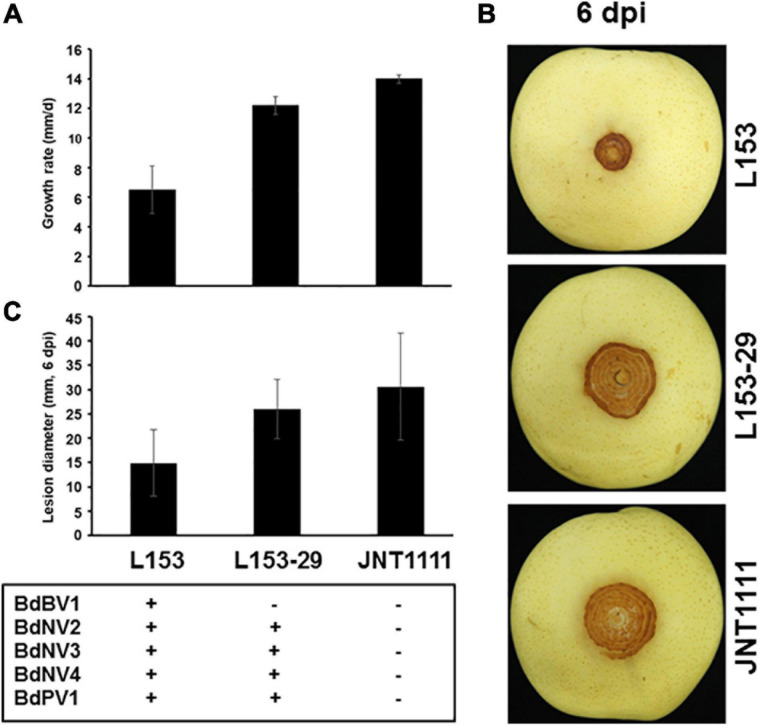
Effects of BdBV1 infection on fungal growth and virulence. **(A)** Growth rates of strains L153, L153-29, and JNT1111 on PDA 25°C. **(B)** Pear fruits (*P. Brettschneider* cv. Huang guan) wound-inoculated with colonized plugs of fungal strains. **(C)** The average diameter of lesions induced on pear fruits. The presence of BdBV1, BdNV2–4, and BdPV1 in these isolates is indicated below the histograms. Symbols “+” and “−” represent the presence and absence of these viruses based on the results of RT-PCR amplification and dsRNA electrophoresis.

The biological traits of strain L153-29 were further accessed together with the parent strain and a virus-free strain JNT1111. The results showed that colonies of strains L153-29 and JNT1111 exhibited radial expansion with a growth rate of 12.2 and 14.0 mm/day, respectively, whereas strain L153 demonstrated irregular growth with sectored regions and a growth rate of 6.5 mm/day (Figure 4A). In the late growth period of these strains, L153 had thinner aerial hypha and higher pigment concentration as compared with those of strains L153-29 and JNT1111 (Figure 3A). Moreover, the hyphal tips of strain L153 were dense and with many short branches, while strain L153-29 was more similar to the strain JNT1111, which showed normal hyphal tips (Figure 3B). Pathological tests showed that strains L153 exhibited reduced virulence (Figure 4B), resulting in a lesion length of 14.9 mm on detached pear fruits 6 dpi (Figure 4C), compared with strains L153-29 and JNT1111, which elicited lesion lengths of 26.0–30.6 mm on the fruits. The results suggest that BdBV1 is responsible for hypovirulence of *B. dothidea* strain L153.

### Structure of Virus Particles Isolated From *Botryosphaeria dothidea* Isolate L153 and Peptide Mass Fingerprinting Analysis of the Proteins

**FIGURE 5:**
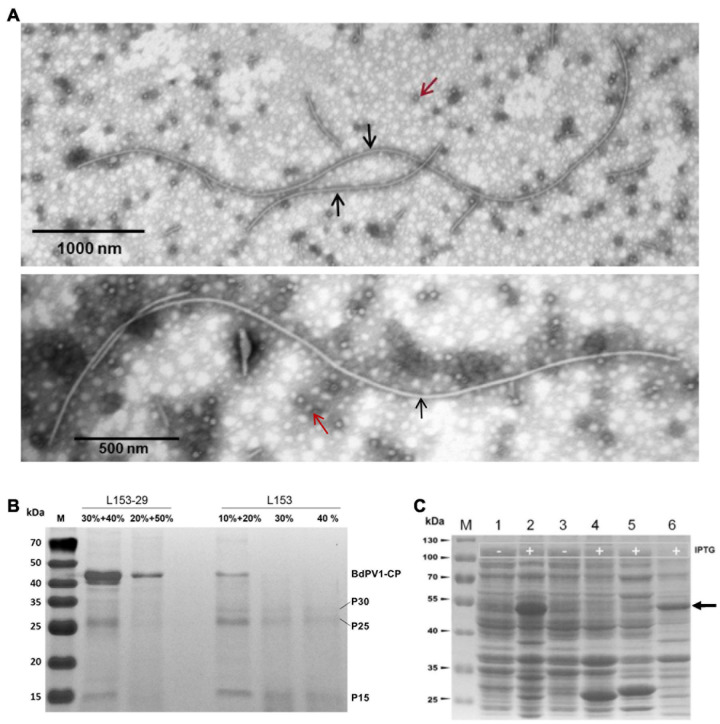
Transmission electron microscopy (TEM) analysis of virus particles and SDS-PAGE analysis of viral and prokaryotic expressed fusion proteins using 12% gel. **(A)** TEM images of virus particles purified from strain L153. Two types of isometric and filamentous particles were observed and indicated by red and black arrows, respectively. **(B)** SDS-PAGE analysis of virus preparations in different sucrose gradients of strains L153-29 and L153. **(C)** SDS-PAGE analysis of expression of fusion proteins in *E. coli* was induced at 28°C overnight. Lines 1 and 2, uninduced and induced *E. coli* transformed with plasmid pGEX-BdBV1-ORF5. Lanes 3 and 4, uninduced and induced *E. coli* transformed with plasmid plasmid pGEX-KG. Lane 4, induced *E. coli* transformed with plasmid pGEX-KG. Lanes 5 and 6, supernatant and precipitated proteins of induced *E. coli* transformed with plasmid pGEX-BdBV1-ORF5 after sonication. Target protein band was indicated by black arrow.

The individual viruses constituting the virome of *B. dothidea* strain L153 were purified and fractionated as described above and examined by TEM. A mixture of isometric and filamentous virus-like particles was observed. Since narnaviruses are unencapsidated (Hillman and Cai, 2013), the two types of virus particles observed are assumed to correspond to BdPV1 and BdBV1 (Figure 5A), respectively. A total of 54 filamentous particles were selected and measured, and had lengths ranging from 760.0 to 5,451.5 nm with diameters ranging from 16.0 to 22.8 nm (Supplementary Table 2). Viral proteins purified from strain L153-29 (BdBV1-free) and L153 (BdBV1-infected) were subjected to SDS-PAGE analysis. The results showed the presence of four proteins in the former (Figure 5B). Two of the proteins 40–50 kDa in size are assumed to be the BdPV1 structural proteins (Wang et al., 2014). Another three proteins named P30, P25, and P15 based on their MW were identified, of which P30 was only detected in strain L153.

To further identify virus-specific proteins, PMF was used and revealed that P30, P25, and P15 generated a total of 7, 20, and 10 peptide fragments, respectively (Supplementary Table 3). Of these fragments, the sequences of the P30 fragments matched the deduced BdBV1 CP sequence at aa position 72–222, accounting for 38% of the entire coverage, suggesting that P30 is most likely the viral CP, P5 encoded by BdBV1 ORF5. The sequences of the P25 and P15 fragments all matched the BdPV1 CP sequence.

### Immunosorbent Electron Microscopy Analysis

**FIGURE 6:**
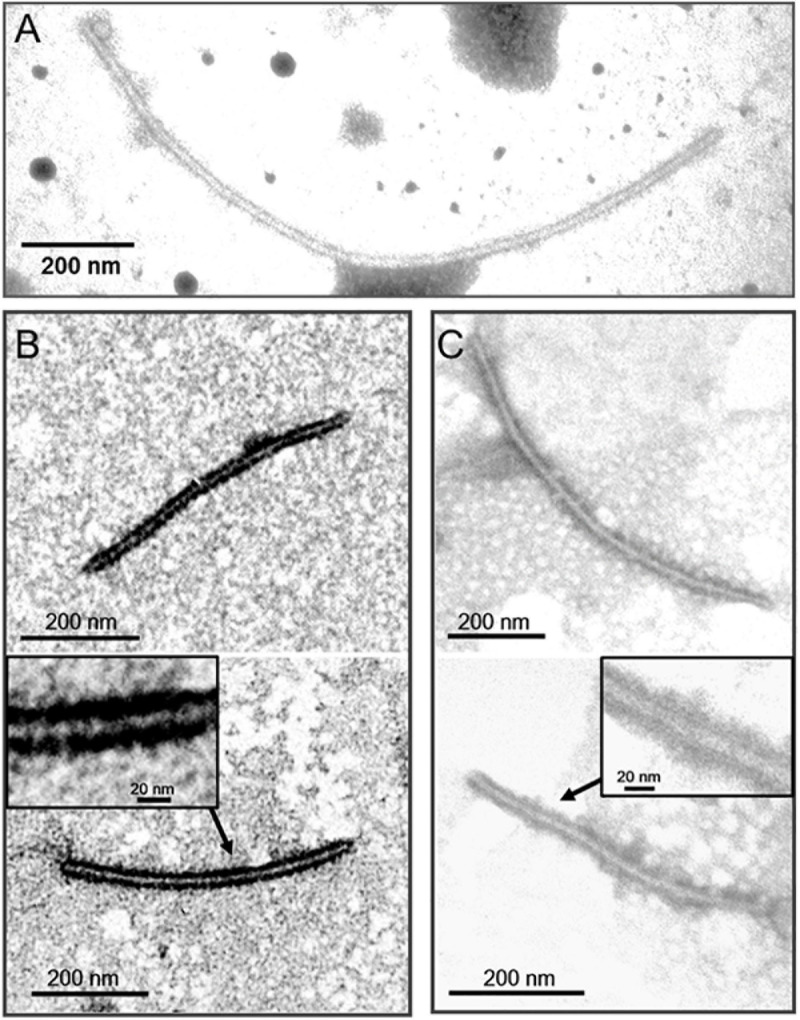
Immunosorbent electron microscopy (ISEM) analysis using PAb-P5 in 4,000- **(A,C)** or 2,000-fold **(B)** dilution. The areas marked by arrow were enlarged in black frame.

To determine if P5 coats the virus as a structural protein, the BdBV1 p5 ORF was expressed *in vitro* (Figure 5C) and used to raise an antibody (pAb-p5) in rabbits following PAGE purification of the expressed protein. Immunoblot analysis revealed that PAb-P5 specifically recognized the P5 protein in three sucrose gradient fractions of virus purified from strain L153 in, i.e., 10, 20% (Supplementary Figure 3A), and 30% (line 4) sucrose but not from preparations of the BdBV1-free, L153-29 isolate (lines 1 and 2). These observations suggest that PAb-P5 specifically recognizes BdBV1 particles. ELISA analysis showed that PAb-P5 had better immune antigenicity between 1,000 and 4,000-fold dilution (Supplementary Figure 3B). Moreover, when ISEM was performed, the results revealed that filamentous particles were clearly decorated with PAb-P5 (Figures 6A–C). The results illustrate that BdBV1 is encapsidated by a CP in filamentous particles in association with an accessory P5 protein.

## Discussion

In this study, we have isolated and sequenced a novel botrexvirus from the phytopathogenic fungus *B. dothidea*, which has been named BdBV1. BdBV1 contains two 3′-co-terminal + ssRNA genomes (RNA1 and RNA2), both of which had high GC contents of 62.5 and 63.7%, respectively. Moreover, 248 nt of the BdBV1 RNA2 3′ terminus was completely consistent with the corresponding position on BdBV1 RNA1, which is characteristic for segmented viruses (Sato et al., 2018). This feature indicates that both RNAs constitute the genome of the same virus and that RNA2 is not a subgenomic RNA derived from RNA1. We further demonstrated by RACE and RT-PCR amplification that the two RNAs exist independently. It is worthy to note that all known alphaflexiviruses, no matter whether they were isolated from fungal or plant hosts, contain a large monopartite + ssRNA genome, whereas BdBV1 appears to be uniquely bi-segmented. Our study showed that the BdBV1 genome may have originated from non-segmented genomes harbored by other members in this family as based on genome comparisons and phylogenetic analyses. These observations might explain why most dsRNA mycoviruses are segmented, while + ssRNA fungal viruses are more likely to be non-segmented. These observations consider that dsRNA viruses may have repeatedly originated from distinct supergroups of (+)RNA viruses (Koonin et al., 2015). Recent investigations have reported that dsRNA viruses with 8 and 11 dsRNA segments were phylogenetically more closely related to + ssRNA virus groups (Jia et al., 2017; Sato et al., 2020).

*Botryosphaeria dothidea* botrexvirus 1 P1 shared the highest aa sequence identities with the replicases of botrexviruses and allexiviruses in the family *Alphaflexiviridae*. The BdBV1 CP sequence showed highest identities to potexviruses and botrexviruses. This observation suggests that the BdBV1 genome is related to the both fungal and plant viruses. Previous investigations have revealed that the majority of mycovirus families may contain members from plant hosts as exemplified by deltapartitiviruses in the family *Partitiviridae* (King et al., 2018; Kamitani et al., 2020), alphachrysoviruses in the family *Chrysoviridae*, and ourmiaviruses in the family *Botourmiaviridae* (Ayllón et al., 2020; Kotta-Loizou et al., 2020), and some families, which are representatives from both fungal and plant viruses (Beever et al., 2001; Howitt et al., 2006; Xie et al., 2006). The BdBV1 genome sequence might represent an example of an evolutionary link between plant and fungal viruses. Multipartite viruses containing segmented and shorter genomic components might benefit from viral replication and prevent adverse mutations (Szathmáry, 1992; Mahy, 2010; Samuel et al., 2011). The BdBV1 genome and the only other botrexvirus genome, BotVX, encode five ORFs. A comparison of the lengths of the RNA1 elements in the family *Alphaflexiviridae* shows that BdBV1 is the shortest (Supplementary Figure 4). Three of the BdBV1 ORFs (ORF2–4) encode putative proteins, which share no detectable similarities with known viral proteins. However, BdBV1 ORF3 shares a low identity with a putative protein from *C. neoformans*, suggesting that horizontal gene transmission may have occurred between BdBV1 and its host (Moreira and López-García, 2009; Liu et al., 2010; Chiba et al., 2011), but this requires further investigation. Nevertheless, BdBV1 has a unique genomic organization compared with BotVX and other types of species from different genera in the family *Alphaflexiviridae*. This property might be a reflection of a gene transfer event, which is widespread in multipartite viruses (Chare and Holmes, 2006; Lefeuvre et al., 2008).

Although *B. dothidea* strain L153 multiplies infected with five mycoviruses (BdBV1, one partitivirus, and three narnaviruses), filamentous BdBV1 particles are easily distinguishable from the partitivirus and narnaviruses, which are, respectively, encapsidated in isometric particles or naked (Hillman and Cai, 2013; Wang et al., 2014). This result was confirmed by ISEM. SDS-PAGE analysis revealed that BdBV1 CP migrated as an ∼30-kDa protein (Figure 5B), which was significantly larger than the predicted MW of 25 kDa, possibly due to its hydrophobic properties, as described in shallot virus X (Kanyuka et al., 1992; Kreuze et al., 2012). Known members of the family *Alphaflexiviridae* possess flexuous filamentous virions that are 470–800 nm in length with a diameter of 12–13 nm (Kreuze et al., 2012). However, BdBV1 differs and is significantly longer and wider than typical alphaflexiviruses (Supplementary Table 1).

This study reports a novel bipartite botrexvirus closely related to fungal and plant viruses, mono- and multi-partite viruses, and contributes useful information on better understanding virus evolution. This study also presents novel molecular traits for an *Alphaflexivirus*, and the first virus particles characterized for a mycovirus under the *Alphaflexiviridae* and contributes new knowledge to the family.

## Data Availability Statement

The datasets presented in this study can be found in online repositories. The names of the repository/repositories and accession number(s) can be found in the article/Supplementary Material.

## Ethics Statement

The animal study was reviewed and approved by the Research Ethics Committee, Huazhong Agricultural University, Hubei, China, and carried out in accordance with the recommendations in the Guide for the Care and Use of Laboratory Animals from this Committee.

## Author Contributions

MY performed the experiments and wrote the manuscript. WX revised the manuscript. XZ selected the manuscript format and corrected the references. ZY provided the NGS analysis. YW provided the PGEX-KG vector. FX and YG collected the fungal strain L153. NH was involved in the design of the investigation. GW designed and supervised the investigation. All authors contributed to the article and approved the submitted version.

## Conflict of Interest

The authors declare that the research was conducted in the absence of any commercial or financial relationships that could be construed as a potential conflict of interest.

## Footnotes

1 https://blast.ncbi.nlm.nih.gov/Blast.cgi

2 https://www.ebi.ac.uk/Tools/msa/mafft/

3 http://www.ncbi.nlm.nih.gov/genomes

## Supplementary Material

Supplementary Figure 1 Detection of BdBV1, BdNV2–4, and BdPV1 of *B. dothidea* strains. **(A)** Agarose gel electrophoresis of dsRNA extracted from strain L153-29. **(B)** RT-PCR detection of BdBV1 and three narnaviruses in *B. dothidea* strains. Each virus is detected with two sets of primers, which are indicated above and below, respectively.

Supplementary Figure 2 Phylogenetic tree based on the amino acid sequences of RdRp of BdNV2–4 and its related viruses. BdNV2–4 are indicated by black circle.

Supplementary Figure 3 Immunoblot analysis and titer quantification of a polyclonal antibody against BdBV1 CP. **(A)** Immunoblot of viral preparations in different sucrose gradients of strain L153-29 and L153 using the antibody against BdBV1 CP (PAb-P5). **(B)** Titer quantification of PAb-P5 by indirect ELISA against the CP from viral preparations using PAb-P5 diluted from 500- to 512,000-fold. P/N, ratio of the absorbance values of the positive sample (BdBV1-infected strain L153) against the negative sample (control strain JNT1111) at a wavelength of 450 nm.

Supplementary Figure 4 Genome comparisons of BdBV1 and type species of different genera in the family *Alphaflexiviridae*. Block regions represent replicase (blue), movement (yellow), coat (red), nucleic acid binding (gray), and unknown (white) proteins.
