# Supplementary material for: Discovery and Characterization of a Novel Bipartite Botrexvirus From the Phytopathogenic Fungus Botryosphaeria dothidea
Source: Front Microbiol. 2021 Jul 1;12:696125. doi: 10.3389/fmicb.2021.696125 (PMC8280476; doi:10.3389/fmicb.2021.696125)
Supplement: Supplementary Figure 1 — Detection of BdBV1, BdNV2–4, and BdPV1 of B. dothidea strains. (A) Agarose gel electrophoresis of dsRNA extracted from strain L153-29. (B) RT-PCR detection of BdBV1 and three narnaviruses in B. dothidea strains. Each virus is detected with two sets of primers, which are indicated above and below, respectively. [file Data_Sheet_1.ZIP › Supplementary Tables.docx]

**Table S1** Information of filamentous/rod shaped +ssRNA viruses

| **Family/virus** | **Hose range** | **No. of segments** | **Genome size (kb)** | **Morphology** | **Virion size (nm)** |
| --- | --- | --- | --- | --- | --- |
| **BdBV1** | Fungi | 2 | 6.1 | Filamentous | 19×>5400* |
| ***Alphaflexiviridae*** | Plants and fungi | 1 | 5.5-9 | Flexuous filamentous | 12-13×470-800 |
| ***Betaflexiviridae*** | Plants | 1 | 5.9-9 | Flexuous filamentous | 10-15×600-1000 |
| ***Gammaflexiviridae*** | Fungi | 1 | 6.8 | Flexuous filamentous | 13×720 |
| ***Benyviridae*** | Plants | 2 | 11.6 | Rod shaped | 20×390 |
| ***Closteroviridae*** | Plants | 1-3 | 13-19.3 | Flexuous filamentous | 12×650-2200 |
| ***Potyviridae*** | Plants | 1 or 2 | 8-11 | Flexuous filamentous | 11-20×680-900 |
| ***Roniviridae*** | Animal | 1 | 26-27 | Bacilliform or rod shaped | 40-60×150-200 |
| ***Virgaviridae*** | Plants | 1-3 | 6.3-13 | Rod shaped | 20-300 |

*The average diameter of the virus particles observed in Table S1 is 19 nm

**Table S2** Length and diameter of 54 selected filamentous virions

| No. | Length (nm) | Diameter (nm) | No. | Length (nm) | Diameter (nm) |
| --- | --- | --- | --- | --- | --- |
| **1** | 21.9 | 5451.5 | **28** | 19.3 | 1765.0 |
| **2** | 19.3 | 5188.6 | **29** | 19.6 | 1725.2 |
| **3** | 21.3 | 4866.5 | **30** | 17.6 | 1604.3 |
| **4** | 22.5 | 3977.7 | **31** | 18.6 | 1545.4 |
| **5** | 18.7 | 3900.5 | **32** | 21.7 | 1504.7 |
| **6** | 19.2 | 3605.7 | **33** | 18.9 | 1466.0 |
| **7** | 16.7 | 3418.1 | **34** | 18.7 | 1420.8 |
| **8** | 16.7 | 3042.6 | **35** | 18.0 | 1399.3 |
| **9** | 18.7 | 2917.3 | **36** | 17.8 | 1387.4 |
| **10** | 16.7 | 2748.1 | **37** | 17.6 | 1363.7 |
| **11** | 18.6 | 2582.7 | **38** | 19.8 | 1328.4 |
| **12** | 22.5 | 2555.7 | **39** | 22.8 | 1307.0 |
| **13** | 20.3 | 2479.4 | **40** | 20.3 | 1284.8 |
| **14** | 22.3 | 2446.0 | **41** | 17.9 | 1255.6 |
| **15** | 19.0 | 2371.2 | **42** | 20.2 | 1189.6 |
| **16** | 21.5 | 2356.7 | **43** | 19.0 | 1140.8 |
| **17** | 17.2 | 2342.5 | **44** | 21.4 | 1111.0 |
| **18** | 18.8 | 2317.7 | **45** | 20.6 | 1109.9 |
| **19** | 18.3 | 2258.4 | **46** | 17.7 | 1101.7 |
| **20** | 19.4 | 2192.5 | **47** | 18.0 | 1079.8 |
| **21** | 18.8 | 2107.1 | **48** | 16.0 | 1057.1 |
| **22** | 17.2 | 2023.2 | **49** | 18.8 | 1019.3 |
| **23** | 16.7 | 2004.3 | **50** | 17.4 | 972.1 |
| **24** | 17.6 | 1938.6 | **51** | 18.8 | 949.8 |
| **25** | 19.5 | 1852.5 | **52** | 21.2 | 919.2 |
| **26** | 21.3 | 1828.9 | **53** | 21.3 | 910.5 |
| **27** | 19.2 | 1824.6 | **54** | 20.4 | 760.0 |

**Table S3** Summary of the peptide mass fingerprinting (PMF) analysis of P30, P25 and P15

| Amino position | Acid Caculated Mass | Observed Mass | Expect | Miss | Amino acid sequence | Ions score |
| --- | --- | --- | --- | --- | --- | --- |
| **P30** |  |  |  |  |  |  |
| 72-86 | 1555.7994 | 778.9077 | 1.6e-006 | 0 | DLDPNPAGLAYALAR | 74 |
| 99-105 | 787.4228 | 394.7193 | 0.0051 | 0 | LEGVWGK | 39 |
| 106-119 | 1582.9307 | 396.7406 | 0.00052 | 0 | KPTPLQHAIPVVQR | 41 |
| 133-140 | 961.4691 | 481.7397 | 0.039 | 0 | AVWNDMVK | 32 |
| 143-150 | 911.4613 | 456.7376 | 0.0071 | 0 | TPPANWAR | 26 |
| 188-202 | 1630.8427 | 816.4287 | 8.8e-006 | 0 | ELQGAQLNAAYAINR | 56 |
| 203-222 | 2240.0682 | 561.0256 | 0.59 | 1 | SREETVHSTNAHATFAAQQR | 5 |
| **P25** |  |  |  |  |  |  |
| 2-24 | 2359.0598 | 787.3611 | 0.00011 | 0 | SGKPTDTNTMHGDSAPASTEAVK | 53 |
| 2-29 | 2883.3192 | 721.8384 | 0.0017 | 1 | SGKPTDTNTMHGDSAPASTEAVKSDPPK | 44 |
| 50-78 | 2976.3949 | 993.1425 | 9.1e-006 | 1 | TESSNSKSTPGFSEGTSVLQNYGLSVSGR | 54 |
| 57-78 | 2242.0866 | 1122.0487 | 9.9e-009 | 0 | STPGFSEGTSVLQNYGLSVSGR | 86 |
| 57-79 | 2370.1816 | 791.0685 | 0.21 | 1 | STPGFSEGTSVLQNYGLSVSGRK | 10 |
| 82-87 | 703.4268 | 352.7196 | 0.0012 | 0 | ILSPFK | 38 |
| 88-101 | 1795.8682 | 599.6309 | 0.0014 | 1 | ADKFFHFVEQSWQK | 36 |
| 91-101 | 1481.7092 | 494.9114 | 0.0079 | 0 | FFHFVEQSWQK | 28 |
| 102-112 | 1208.7241 | 403.9148 | 3.5e-005 | 0 | LVSVKPSLPNR | 53 |
| 113- 19 | 884.4392 | 443.2268 | 0.00091 | 0 | FSLAEYR | 49 |
| 120-129 | 1185.6254 | 396.2172 | 0.002 | 0 | HASALQLYQR | 41 |
| 130-147 | 1928.0731 | 483.0261 | 4.4e-009 | 1 | IEAVKFDSLGIKPSAPTR | 91 |
| 135-147 | 1387.7460 | 463.5889 | 2.8e-007 | 0 | FDSLGIKPSAPTR | 66 |
| 210-219 | 1162.5618 | 582.2833 | 6.3e-008 | 0 | SSWEDVLTAR | 83 |
| 320-336 | 2095.0109 | 699.3484 | 6.6e-005 | 1 | LKSPQDYAEEIEELFER | 49 |
| 322-336 | 1853.8319 | 618.9526 | 1.3e-005 | 0 | SPQDYAEEIEELFER | 62 |
| 349-375 | 2812.2828 | 938.4365 | 3.9e-005 | 0 | FDVSYQSSSYVVSDGTITADPGAYGAR | 50 |
| 434-457 | 2606.4319 | 869.8157 | 6.9e-007 | 0 | ASIPPATWVLSLLLQSSTLPEDLR | 70 |
| 458-474 | 1855.9316 | 928.9746 | 9.6e-008 | 0 | STFFTETDGLSNVLGLR | 86 |
| 484-494 | 1264.5976 | 633.3075 | 0.0004 | 0 | TPPPIEQYGTY | 54 |
| **P15** |  |  |  |  |  |  |
| 57-78 | 2242.0866 | 748.3692 | 2.9e-007 | 0 | STPGFSEGTSVLQNYGLSVSGR | 76 |
| 57-79 | 2370.1816 | 791.0667 | 1 | 1 | STPGFSEGTSVLQNYGLSVSGRK | 14 |
| 88-101 | 1795.8682 | 449.9736 | 1 | 1 | ADKFFHFVEQSWQK | 11 |
| 91-101 | 1481.7092 | 494.9097 | 0.11 | 0 | FFHFVEQSWQK | 21 |
| 102-112 | 1208.7241 | 403.9144 | 5.5e-006 | 0 | LVSVKPSLPNR | 57 |
| 113-119 | 884.4392 | 443.2256 | 0.0012 | 0 | FSLAEYR | 48 |
| 120-129 | 1185.6254 | 396.2156 | 0.00012 | 0 | HASALQLYQR | 48 |
| 135-147 | 1387.7460 | 463.5882 | 2.8e-007 | 0 | FDSLGIKPSAPTR | 74 |
| 458-474 | 1855.9316 | 619.6506 | 7.7e-010 | 0 | STFFTETDGLSNVLGLR | 105 |
| 484-494 | 1264.5976 | 633.3048 | 0.0081 | 0 | TPPPIEQYGTY | 40 |

**Table S4** Information on the virus isolates used for sequence alignment, phylogenetic analysis and genome comparison**^*^**

| Family and Genus | Virus | Abbreviation | Accession no. | |
| --- | --- | --- | --- | --- |
|  |  |  | Replicase | CP |
| ***Alphaflexiviridae*** |  |  |  |  |
| *Allexivirus* | Garlic virus A | GarV-A | NP_569126.1 | NP_569130.1 |
|  | Garlic virus B | GarV-B | YP_009110668.1 | YP_009110672.1 |
|  | Garlic virus C | GarV-C | NP_569132.1 | NP_569136.1 |
|  | Garlic virus X | GarV-X | NP_044571.1 | NP_044575.1 |
|  | Cassia mild mosaic virus | CasMMV | ADD65542.1 | -- |
|  | Shallot virus X | ShVX | NP_620648.1 | NP_620652.1 |
|  | Alfalfa virus S | AVS | YP_009362668.1 | YP_009362673.1 |
|  | Arachis pintoi virus | ApV | -- | YP_009328896.1 |
|  | Blackberry virus E | BVE | YP_004659200.1 | YP_004659204.1 |
|  | Vanilla latent virus | VLV | YP_009389473.1 | YP_009389478.1 |
| *Botrexvirus* | Botrytis virus X | BotVX | NP_932306.1 | NP_932309.1 |
| *Lolavirus* | Lolium latent virus | LoLV | YP_001718499.1 | YP_001718503.1 |
| *Potexvirus* | Cymbidium mosaic virus | CymMV | NP_054025.1 | NP_054029.1 |
|  | Yam virus X | YVX | YP_009091814.1 | YP_009091818.1 |
|  | Bamboo mosaic virus | BaMV | -- | NP_042587.1 |
|  | Nerine virus X | NVX-J | -- | YP_446996.1 |
|  | Strawberry mild yellow edge virus | SMYEV | NP_620642.1 | NP_620646.1 |
|  | Lily virus X | LVX | YP_263303.1 | -- |
|  | Potato virus X | PVX | YP_002332929.1 | YP_002332933.1 |
|  | White clover mosaic virus | WCMV | NP_620715.1 | NP_620719.1 |
| *Mandarivirus* | Indian citrus ringspot virus | ICRSV | NP_203553.1 | NP_203557.1 |
|  | Citrus yellow vein clearing virus | CYVCV | YP_009124988.1 | YP_009124992.1 |
| *Platypuvirus* | Donkey orchid symptomless virus | DOSV | YP_008828152.1 | YP_008828154.1 |
| *Sclerodarnavirus* | Sclerotinia sclerotiorum debilitation-associated RNA virus | SsDRV | YP_325662.1 | -- |
| ***Betaflexiviridae*** |  |  |  |  |
| *Capillovirus* | Apple stem grooving virus | ASGV | -- | AAL40796.1 |
| *Trichovirus* | Apple chlorotic leaf spot virus | ACLSV | -- | CAB46654.1 |
| *Vitivirus* | Grapevine virus A | GVA | -- | AAL76173.1 |
| *Foveavirus* | Grapevine virus T | GVT | -- | YP_009389472.1 |
|  | Panax ginseng flexivirus 1 |  | YP_009552761.1 | -- |
| ***Gammaflexiviridae*** |  |  |  |  |
| *Mycoflexivirus* | Botrytis virus F | BotVF | NP_068549.1 | NP_068550.1 |

**^*^**The phylogenetic analyses were shown in Fig. 2, the sequence alignments were presented in Fig. 1 and the sequence comparison is shown in Supplementary Fig. 3.
