# Supplementary figures and images for: Discovery and Characterization of a Novel Bipartite Botrexvirus From the Phytopathogenic Fungus Botryosphaeria dothidea
Source: Front Microbiol. 2021 Jul 1;12:696125. doi: 10.3389/fmicb.2021.696125 (PMC8280476; doi:10.3389/fmicb.2021.696125)

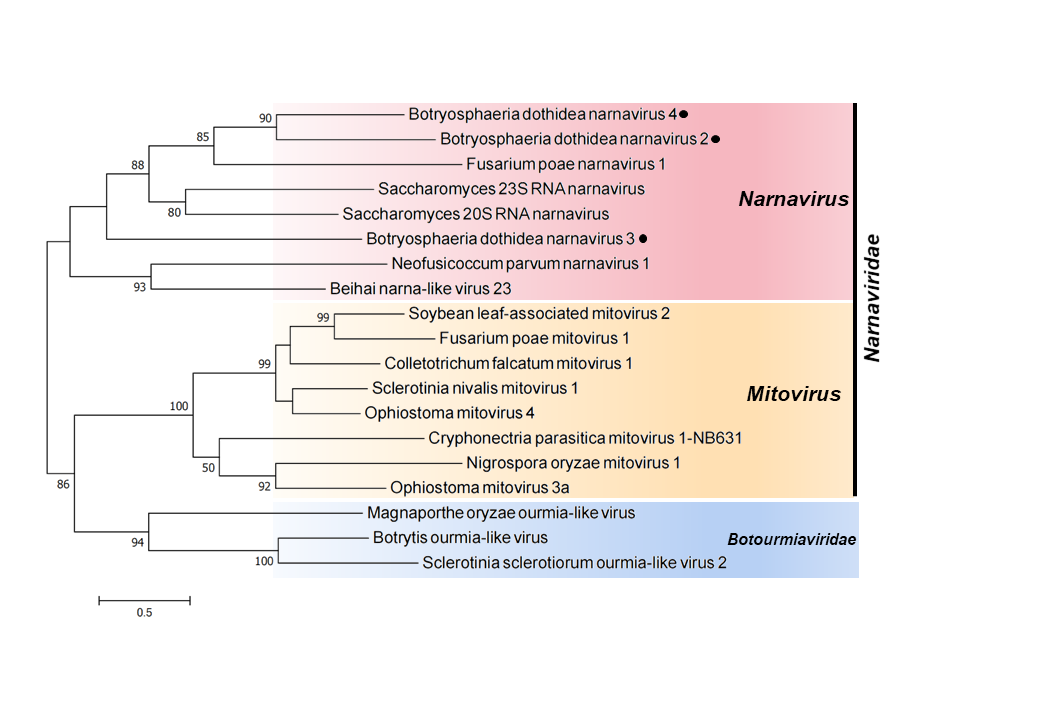

Supplement: Supplementary Figure 1 — Detection of BdBV1, BdNV2–4, and BdPV1 of B. dothidea strains. (A) Agarose gel electrophoresis of dsRNA extracted from strain L153-29. (B) RT-PCR detection of BdBV1 and three narnaviruses in B. dothidea strains. Each virus is detected with two sets of primers, which are indicated above and below, respectively. [file Data_Sheet_1.ZIP › Fig S2.tif]

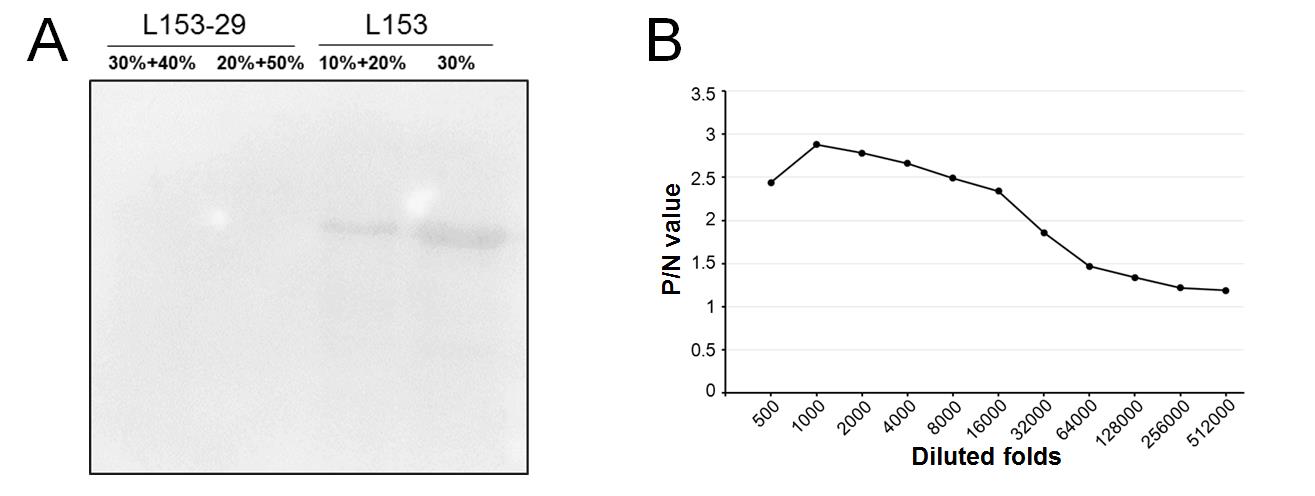

Supplement: Supplementary Figure 1 — Detection of BdBV1, BdNV2–4, and BdPV1 of B. dothidea strains. (A) Agarose gel electrophoresis of dsRNA extracted from strain L153-29. (B) RT-PCR detection of BdBV1 and three narnaviruses in B. dothidea strains. Each virus is detected with two sets of primers, which are indicated above and below, respectively. [file Data_Sheet_1.ZIP › Fig S3.jpg]

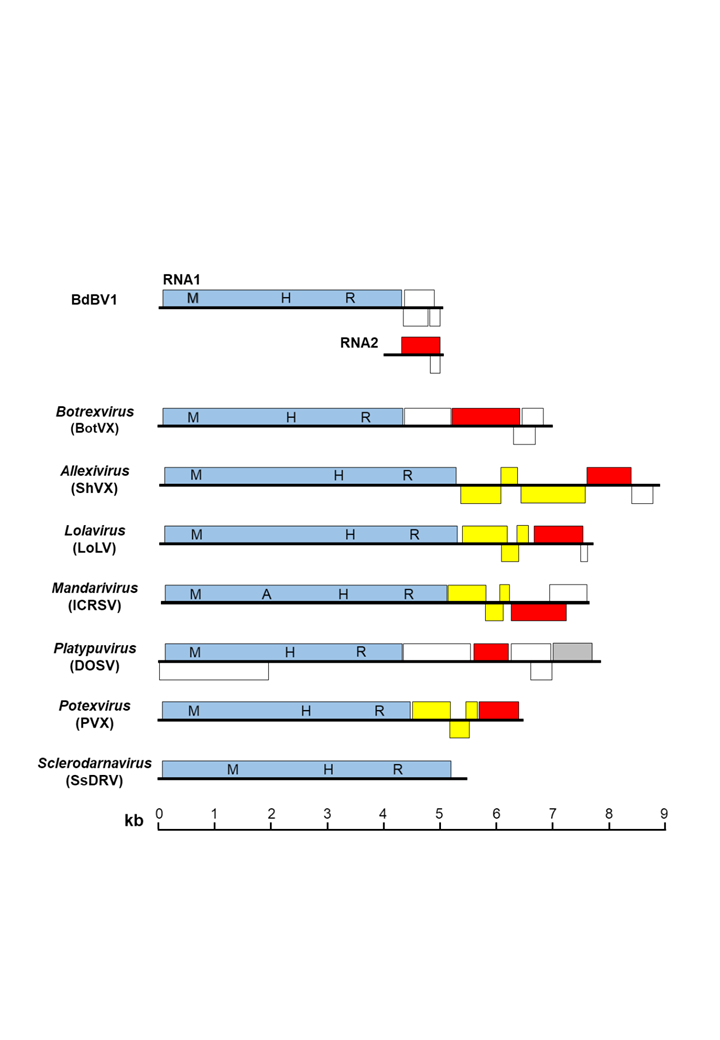

Supplement: Supplementary Figure 1 — Detection of BdBV1, BdNV2–4, and BdPV1 of B. dothidea strains. (A) Agarose gel electrophoresis of dsRNA extracted from strain L153-29. (B) RT-PCR detection of BdBV1 and three narnaviruses in B. dothidea strains. Each virus is detected with two sets of primers, which are indicated above and below, respectively. [file Data_Sheet_1.ZIP › Fig S4.tif]

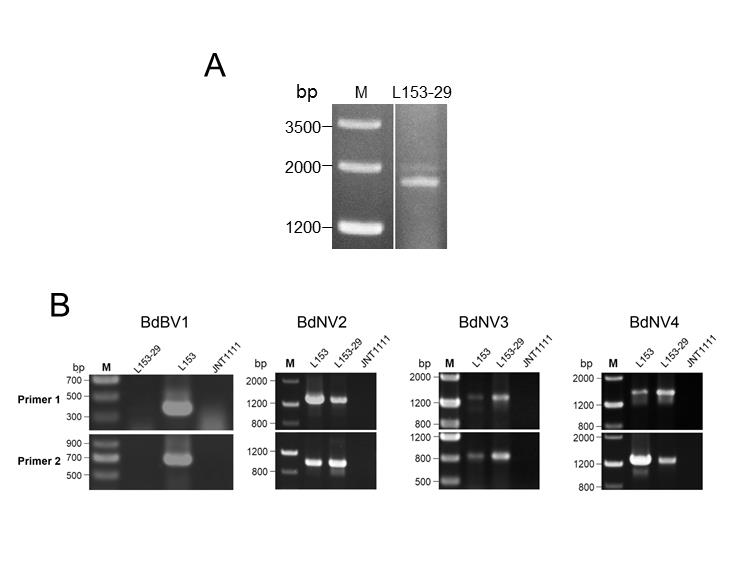

Supplement: Supplementary Figure 1 — Detection of BdBV1, BdNV2–4, and BdPV1 of B. dothidea strains. (A) Agarose gel electrophoresis of dsRNA extracted from strain L153-29. (B) RT-PCR detection of BdBV1 and three narnaviruses in B. dothidea strains. Each virus is detected with two sets of primers, which are indicated above and below, respectively. [file Data_Sheet_1.ZIP › Fig S1.jpg]
